# Food and Beverage Cues Featured in YouTube Videos of Social Media Influencers Popular With Children: An Exploratory Study

**DOI:** 10.3389/fpsyg.2019.02142

**Published:** 2019-09-20

**Authors:** Anna E. Coates, Charlotte A. Hardman, Jason C. G. Halford, Paul Christiansen, Emma J. Boyland

**Affiliations:** Department of Psychological Sciences, Institute of Population Health Sciences, University of Liverpool, Liverpool, United Kingdom

**Keywords:** food cue, beverage cue, YouTube, influencer, social media, advertising, marketing, children

## Abstract

Food and beverage cues (visual displays of food or beverage products/brands) featured in traditional broadcast and digital marketing are predominantly for products high in fat, sugar and/or salt (HFSS). YouTube is hugely popular with children, and cues featured in content uploaded by YouTube video bloggers (influencers) has been shown to affect children’s eating behavior. However, little is known about the prevalence of such cues, the contexts in which they appear, and the frequency with which they are featured as part of explicit marketing campaigns. The objective of this study was to explore the extent and nature of food and beverage cues featured in YouTube videos of influencers popular with children. All videos uploaded by two influencers (one female, one male) over a year (2017) were analyzed. Based on previous content analyses of broadcast marketing, cues were categorized by product type and classified as “healthy” or “less healthy” according to the UK Nutrient Profiling Model. Cues were also coded for branding status, and other factors related to their display (e.g., description). In total, the sample comprised 380 YouTube videos (119.5 h) and, of these, only 27 videos (7.4%) did not feature any food or beverage cues. Cakes (9.4%) and fast foods (8.9%) were the most frequently featured product types, less frequent were healthier products such as fruits (6.5%) and vegetables (5.8%). Overall, cues were more frequently classified as less healthy (49.4%) than healthy (34.5%) and were presented in different contexts according to nutritional profile. Less healthy foods (compared with healthy foods) were more often; branded, presented in the context of eating out, described positively, not consumed, and featured as part of an explicit marketing campaign. These data provide the first empirical assessment of the extent and nature of food and beverage cue presentation in YouTube videos by influencers popular with children. Given the emerging evidence of the effects of influencer marketing of food and beverages on children’s eating behavior, this exploratory study offers a novel methodological platform for digital food marketing assessment and delivers important contextual information that could inform policy deliberations in this area.

## Introduction

The global rise in childhood obesity rates over the past few decades is at least partly due to changes in the food environment (Swinburn et al., 2011; Wang and Lim, 2012; Powell et al., 2013a; Ng et al., 2014). There is robust evidence that children’s exposure to marketing of food and beverages high in fat, sugar and/or salt (HFSS) contributes to these increasing levels of obesity (Norman et al., 2016; WHO, 2017). While numerous studies have explored the prevalence of food and beverage marketing in broadcast media (Kelly et al., 2019), prevalence in digital media where marketing is predominately targeted and personalized is more challenging to measure. Social media, in particular YouTube, is hugely popular with children (aged 5–15 years), who report watching content by YouTube video bloggers (Ofcom, 2018). These individuals are often referred to as “influencers” due to the persuasive effect their opinions can have on their audiences (Berryman and Kavka, 2017). Exposure to HFSS food and beverage cues featured in influencers’ social media content have been shown to affect children’s (9–11 years) immediate brand choice and consumption (Coates et al., 2019a, b). Given these cued consumption effects, which mirror those previously found for both television and internet advergaming (Boyland et al., 2016; Folkvord and van ‘t Riet, 2018; Russell et al., 2018) there is a clear need for tools that can effectively quantify the extent and nature of digital marketing, including techniques such as product placement in user-generated social media content (WHO, 2019). This will facilitate a better understanding of children’s likely exposure, and the persuasive ability of that exposure, which is critical for the development of effective public health policy in this area.

A global study, including data contributed from 22 countries, on the prevalence of television (TV) food and beverage advertising during children’s peak viewing times found that 23% of all advertisements were for food or beverages, and that unhealthy products were promoted four times more than healthier products (Kelly et al., 2019). In the United Kingdom, products are classified as “less healthy” or “healthy” using the UK Nutrient Profiling Model (UKNPM) (UK Department of Health, 2011). Less healthy products are prohibited from being advertised in children’s TV programming or programs likely to be of a “particular appeal” to children under 16 years (Ofcom, 2007). Despite this, research shows that the majority of adverts featured during family TV programs, watched by children in substantial numbers, promote less healthy products (59%), and far fewer promote healthy products (17%) (Obesity Health Alliance, 2017). On average, children in the United Kingdom see 3.5 food and beverage adverts per hour (1.9 for HFSS products specifically) and fast food is the most frequently advertised product (representing 15.4% of all foods advertised) (Whalen et al., 2017). Studies typically show a lack, or complete absence, of adverts promoting fruit or vegetables (Powell et al., 2013b; Whalen et al., 2017), which is inconsistent with national dietary recommendations, but is not surprising given the sizeable budgets of the food industry in comparison to health campaigns (Obesity Health Alliance, 2017).

Studies that have explored product placement (e.g., the paid presence of branded products) in children’s programming and popular movies find food and beverages to be prevalent (Auty and Lewis, 2004; Elsey and Harris, 2015). An analysis of children’s programming on United Kingdom and Irish TV found approximately 14.3 food and beverage placements per hour, which is four times more than the rate of TV advertisements for food and beverages (3.5 per hour) (Scully et al., 2014). These food and beverage product placements were most likely to feature HFSS items (47.5%) which, compared with healthy items, were more frequently presented outside of the home, consumed as snacks rather than as balanced meals, involved characters who were of a healthy weight, and were associated more with positive motivating factors. Therefore, not only do HFSS products dominate this marketing landscape, they are also promoted in different ways to healthier options.

The Reactivity to Embedded Food Cues in Advertising Model (REFCAM) states that the level of processing of a food cue influences the effect of exposure (Folkvord et al., 2016). Whereas TV advertising appears at recognizable intervals within and between programming (Owen et al., 2013), product placement embeds the cues directly into the programming itself. Food cues that are more integrated into media content are thought to be processed with minimal cognitive elaboration (Buijzen et al., 2010; Cauberghe and De Pelsmacker, 2010; Folkvord et al., 2016), meaning children may be less able to recognize that they are being advertised to (Freeman et al., 2007; Rozendaal et al., 2011) and find it more difficult to resist marketing of this nature (Folkvord et al., 2016).

Children (5–15 years) in the United Kingdom now spend more time online than they do watching TV (Ofcom, 2018), meaning exposure to digital marketing has also increased concurrently (WHO, 2019). Food and beverage companies are shifting their advertising spend from TV to digital media in order to reach young people (Powell et al., 2013a). Current self-regulatory rules in the United Kingdom prohibit HFSS food and beverage marketing in digital media targeted at children, or where children make up more than 25% of the audience (ASA, 2017). However, a recent report by the Advertising Standards Authority (ASA), the independent advertising regulator in the United Kingdom, provided insight into the effectiveness of these rules and found in just a 2-week monitoring period, 2.3% (947) of all adverts displayed on websites with child specific appeal, promoted HFSS products (ASA, 2019). In addition, almost all (20 out of 21) of the child specific YouTube channels that were monitored displayed at least one HFSS product advertisement. Thus, even by the regulator’s own admission, these rules appear to be ineffectual in reducing children’s exposure to these products.

As children’s media consumption has changed from traditional spaces (TV) to digital spaces (online games, etc.) and social media, research has continually sought to quantify the marketing taking place in those domains. Some have looked at websites and explored brand cues in advergames (Harris et al., 2012; Folkvord et al., 2017; Folkvord and van ‘t Riet, 2018). However, contemporary digital marketing of HFSS foods and beverages is often targeted and personalized, meaning there are methodological challenges in measuring children’s exposure (WHO, 2019). Despite this, research shows predominant promotion of HFSS products compared with healthier products (Culp et al., 2010; Kent and Pauzé, 2018; Tan et al., 2018). For instance, a content analysis on the most popular websites with children (2–11 years) in Canada conducted over the course of a year found approximately 54 million food advertisements (banner and pop up), most (73.8%) promoted “less healthy” foods (Kent and Pauzé, 2018). A study in Malaysia analyzed advertisements in YouTube videos popular with children and found the most frequently advertised products were food and beverages (38%), most (56.3%) were non-core (broadly unhealthy) items (Tan et al., 2018). Another study in Canada screen-captured children’s (7–11 years) and adolescents’ (12–16 years) personal devices (smartphones or tablets) when accessing favorite social media platforms to examine food and beverage marketing exposure (Potvin Kent et al., 2019). The study revealed that 72% of the sample were exposed to this type of marketing, and fast food (44%) was the most frequently advertised food category. The results of these studies are concerning, given the robust evidence that children’s exposure to marketing of HFSS items contributes to increased consumption of unhealthy foods (Boyland et al., 2016) and greater levels of childhood obesity (Norman et al., 2016; WHO, 2017).

It could be argued that HFSS brands that advertise on social media platforms (e.g., Instagram, YouTube, Twitter) do not target their marketing at children, as most of these sites require users to be 13-years and over. However, children can access social media without having a registered account, by using parents accounts, or by using fake date of births to create their own accounts (Ofcom, 2016). As a result, large numbers of young children are active on these sites and therefore exposed to their marketing content (WHO, 2016). Because of the well documented methodological challenges inherent in quantifying behaviorally and contextually targeted marketing in social media (WHO, 2016; Tatlow-Golden et al., 2017), there are limited studies of this kind. However, those that have explored marketing techniques used by HFSS brands on Instagram found posts that featured “healthier” products to be rare (Ginsberg, 2015; Vassallo et al., 2018). In fact, when healthy products were included in posts, they tended to be peripheral to the HFSS product being promoted (e.g., Nutella chocolate spread pictured with fruit), a technique that has been shown to mislead children in their understanding of nutrition (Bernhardt et al., 2014). Additional findings were that Instagram posts were often brand focused (featuring either a brand name or logo), a technique used in TV advertising, exposure to which has brand specific effects on children’s food intake and preference (Borzekowski and Robinson, 2001; Robinson et al., 2007; Forman et al., 2009; Boyland et al., 2013). Instagram posts also regularly featured consumers (including celebrities) whose lifestyles and values were referenced in images (e.g., image of celebrity eating out). A technique based on the assumption that audiences wish to emulate celebrity lifestyles (Hirschman and Thompson, 1997), and so will form a preference for the celebrity-endorsed brand (Hackley and Hackley, 2015). Indeed, children (10–16 years) have been found to perceive HFSS brands as having positive attributes, due to the desirable traits of an endorser featured in an advertisement (Kelly et al., 2016). Therefore, it is clear that exposure to the persuasive techniques used by HFSS marketers can have an impact on children’s food related behaviors.

Food and beverage marketing shared by peers on social media is considered to have a stronger impact on young people than marketing directly from a brand (Buchanan et al., 2017, 2018). This is likely to reflect the greater familiarity young people have with their peers (Berryman and Kavka, 2017; De Veirman et al., 2017), whose recommendations they are therefore more likely to trust (Chu and Kim, 2011; Kim and Johnson, 2016). Food and beverage brands have capitalized on this type of marketing by “seeding” messages in social networks which are then disseminated amongst peers (Brown et al., 2007). A 2014 campaign by Coca-Cola personalized bottles of Coke by printing peoples’ names on labels. The hashtag “#*ShareaCoke*” encouraged social media users to share images of themselves drinking their named product with other social media users (Mendoza, 2015). As a result of exposure to the campaign, 1.25 million more young people consumed a Coca-Cola during the summer of the campaign, compared with the previous summer, which contributed to an 11% increase in volume of sales for that year (Source: Coca-Cola’s brand health tracker – B3). A difficulty when regulating HFSS food marketing on social media is determining whether the content is commercial or is user-generated (Dunlop et al., 2016). Capitalizing on social media networks in this way enables brands to gain seemingly authentic recommendations from consumers, and a wider reach of the original marketing message (Freeman et al., 2014; Kelly et al., 2015) with relatively less financial investment compared with TV advertising (WHO, 2013).

User-generated media content is created by members of the general public and is considered to not be explicitly part of a marketing campaign (Smith et al., 2012). However, research has found that 18% of children’s overall exposure to food and beverage marketing on social media was via user-generated content, and the most marketed product was fast food (58%) (Potvin Kent et al., 2019). In line with these findings, a Swedish study used a youth-oriented hashtag to explore the content of Instagram posts shared by 14 year-olds (Holmberg et al., 2016). In most of the Instagram posts (85%) adolescents featured food and beverages, the majority were HFSS products (67.7%), far fewer were fruits or vegetables (21.8%). Also, HFSS products are often presented in user-generated content with brand names clearly visible (Ginsberg, 2015; Vassallo et al., 2018), replicating a technique in food and beverage marketing (Cairns et al., 2013). It is unclear in these studies whether the marketing identified was encouraged by food and beverage companies (like in the Coca-Cola campaign). However, research suggests that exposure to food and beverages embedded in seemingly authentic content is associated with more positive attitudes and taste evaluations during later consumption (Coary and Poor, 2016), which is concerning not only because of the volume of exposure, but also because of the techniques that blur the lines between advertising and entertaining content.

YouTube is one of the most popular social media platforms with children, with approximately 72% of 10–12-year-olds in Australia (Baldwin et al., 2018), 80% of 5–15-year-olds in the United Kingdom (Ofcom, 2018), and 85% of 13–17-year-olds in the US (Pew Research Center, 2018) reporting regular use. YouTube video blogs are a user-generated form of online communication that serve to document influencers’ day-to-day lives (Snelson, 2015; Lee and Watkins, 2016). The popularity of influencers has risen exponentially (Hovden, 2013). Qualitative interviews with children (5–15 years) in the United Kingdom have revealed that many report watching this content and regard these individuals as being authentic and relatable (Ofcom, 2018). Para-social interaction is a person’s illusion of a relationship between themselves and a media character (Aladwani, 2014), and may explain why children feel as if they know the influencer on a personal level and are trusting of their opinions (Knoll and Matthes, 2017). Featuring brand recommendations in this content is increasingly common (Kelly et al., 2015; Liljander et al., 2015; The Economist, 2016) and companies offer free products or services, gift cards or money to influencers in exchange for positive social media content (Liljander et al., 2015; De Veirman et al., 2017).

In 2018, marketers reportedly spent over $500 million on influencer marketing, which is predicted to increase to $5–10 billion over the next 5 years (Mediakix, 2018) as it is more commonly used (Ashley and Tuten, 2015; Russell and Rasolofoarison, 2017; Potvin Kent et al., 2019). A study in Canada found that 11% of children’s (7–16 years) food marketing exposure while using social media was via content uploaded by celebrities or influencers (Potvin Kent et al., 2019). In the Netherlands, food and beverage brands were the most commonly reported brands that children (10–13 years) recalled viewing in this content (in comparison with “toy,” “daily care,” and “other” brands) (Folkvord et al., 2019). Consistent with these findings, in 2018, eleven of the sixteen most popular influencers with young people in Norway produced YouTube video content that featured food and beverage marketing, most of the promoted products were HFSS (Norwegian Consumer Council, 2019). These findings are a concern given that children (10–13 years) consider themselves and others affected by the endorsements they view in influencers’ YouTube videos (Folkvord et al., 2019).

Given the popularity of YouTube influencers with children, and the previously noted effects of food and beverage cues featured in this content on children’s immediate intake (Coates et al., 2019a, b), it is important that research explores the prevalence of such cues, and the contexts in which they appear. The primary aim of the current study was to quantify the prevalence of food and beverage cues featured in YouTube videos of social media influencers popular with children. A secondary aim was to determine the proportion of “healthy” and “less healthy” (i.e., HFSS) cues featured in these videos, in accordance with the UKNPM. A final aim was to explore the nature of cue presentation, including branding status, and other factors such as the use of cue descriptors (positive or negative), whether or not the food or beverage item was consumed, and whether it was featured as part of an explicit marketing campaign.

## Materials and Methods

### Sample

YouTube videos uploaded by two influencers (one female – age 29, one male – age 24; both considered by the authors to be a healthy weight) were assessed using content analysis methods adapted from similar studies (Sutherland et al., 2010; Boyland et al., 2011; Scully et al., 2014; Holmberg et al., 2016). At the time of writing each influencer had two YouTube channels, a main channel where video blogs capturing everyday life were uploaded (e.g., an influencer films themselves going to a theme park), and a second channel where more genre specific videos were uploaded (e.g., Q&A videos where the influencer answers questions from their subscribers). The female influencer had approximately 16.8 million subscribers, and the male influencer 9.2 million. Both influencers were popular with children between the age of 5–15 years in the United Kingdom (Childwise, 2016), and were selected for the current study in order to be consistent with those used in two experimental studies investigating the impact of social media influencer food marketing on children’s intake (Coates et al., 2019a, b). The study was not seeking to be exhaustive in covering all influencer content viewed by children (this would not be feasible in a researcher-led design, automated tools would be required to analyze such a large volume of video content). Given that the impact of marketing on behavior is a function of both exposure and power (WHO, 2010), this study focused on influencer content children are likely to be exposed to. Children are known to watch these videos and do not just watch videos that are more specifically targeted at them, on the YouTube Kids app for example (Ofcom, 2018). Neither influencer was known for their food expertise or for blogging about food, but both had previously featured in HFSS food marketing campaigns on social media. Videos uploaded over a full 12-month period (January 1, 2017–December 31, 2017) were analyzed, similar to previous studies (Vassallo et al., 2018). There is some evidence that food and beverage advertising varies by season (Boyland et al., 2011), therefore analyzing video content over the course of a year ensured that a representative sample of products were captured. This study did not involve human subjects and therefore ethical approval was not required.

### Data Coding

#### Nutrient Profiling

A food or beverage cue was defined as a visual display/combined visual and verbal display, of a food or beverage product/brand, and was based on a definition in a similar study (Radnitz et al., 2009). The nutritional information required to correctly classify advertised products as “healthy” or “less healthy” was firstly obtained by consulting company websites or Tesco’s website (the largest supermarket chain in the United Kingdom). Where nutritional information was not available for the featured products a similar product was identified in McCance and Widdowson’s Composition of Foods integrated dataset (Public Health England, 2015). Food and beverage items were exclusively classified as “healthy” or “less healthy” (i.e., HFSS), according to the UKNPM. The UKNPM is an established evidence-based tool that evaluates the nutritional composition of food and beverages by analyzing the healthy components (protein, fiber, and vegetables, fruit and nuts) and the less healthy components (sugars, saturated fats, and salt) per 100 g. If a food produces a score of four or more, or beverage a score of one or more, the item was classified as “less healthy.” Below these scores, items were designated as “healthy.” Items were classified as “miscellaneous” if the nutritional content was not able to be calculated (e.g., product not identifiable from the video due to its packaging, product information not available online, etc.) because without this information the product could not be classified as “healthy” or “less healthy.”

#### Food and Beverage Cue Type

Cues were categorized into one of 25 groups which was performed in line with an established coding scheme of food and beverages featured in adverts (Boyland et al., 2011). Multiple cues within a scene were individually coded. Similarly, for meals, items were coded individually if clearly visible (e.g., a McDonalds Happy Meal would be categorized as “cheeseburger,” “fries,” and “Coca-Cola” if all of those items could be seen in the video). Cues that made multiple appearances in the same video were only recorded once. Prominent cues (e.g., a food in the influencers hand, a beverage placed on a table in front of the influencer) that were not verbally referenced by the influencer were coded. However, peripheral cues (e.g., foods on a supermarket shelf behind the influencer) that were not verbally referenced by the influencer were not coded. It was considered that if attention was not brought to these items by the influencer referring to them then these cues were not prominent. Cues that were verbally referred to by the influencer but were not visually present (e.g., the influencer talks about a craving for McDonalds) were also not coded due to a considered lack of prominence.

#### Food and Beverage Cue Brand

Coding of brands of cues was performed in line with previous research (Sutherland et al., 2010). A branded cue was defined as any food or beverage with an identifiable logo and/or product name. All food or beverage cues were categorized into one of five mutually exclusive groups (food brand, food retail establishment brand, supermarket brand, unbranded, miscellaneous) (see Table 1). If a food brand was shown but no product(s) (e.g., McDonalds golden arches), this was coded as a branded cue. Internet sales data were researched to find the biggest selling product from that brand which was entered as the food or beverage cue.

**TABLE 1:** Food and beverage cue brand and presentation categories and description.

**Category**	**Description of category**
**Cue brand**	
Food and beverage brand	Product brand (e.g., Heinz) is recognizable (i.e., brand icon is visually apparent/brand name is verbally stated)
Food retail establishment brand	Food retail establishment brand (e.g., McDonalds) is recognizable (i.e., brand icon is visually apparent/brand name is verbally stated)
Supermarket-own brand	Supermarket own brand (e.g., Tesco) is recognizable (i.e., brand icon is visually apparent/brand name is verbally stated)
Unbranded	Product is an unbranded item (e.g., bag of loose oranges)
Miscellaneous	Product brand is not recognizable (e.g., hamburger with packaging removed)
**Cue context**	
Eating out meal	Product presented in/purchased as a take-away item in food retail establishment (e.g., fast food restaurant, coffee shop, café)
Supermarket	Product presented in a supermarket (including market, convenience store)
Home	Product presented in the home
Other	Product presented in a context outside of the above categories (e.g., park, car, beach)
**Cue description**	
Positive	Product described using positive adjectives or tone
Negative	Product described using negative adjectives or tone
Neutral	Product described using a neutral adjectives or tone (e.g., equal use of positive and negative adjectives, no adjectives)
**Cue presentation**	
Consumed and verbally referenced	Product consumed and verbal reference made
Consumed and not verbally referenced	Product consumed but no verbal reference made
Not consumed and verbally referenced	Product not consumed, and verbal reference made (i.e., visual and verbal presentation)
Not consumed and not verbally referenced	Product not consumed, and no verbal reference made (i.e., visual presentation only)
**Reason cue was featured**	
Non-marketing	No indication that influencer was gifted or paid to feature product in YouTube video
Gifted endorsement	Gifted endorsement of product (influencer indicated they have been gifted/sent product by brand)
Paid endorsement	Paid endorsement of product (i.e., on-screen advertising disclosure, influencer indicated they were paid to feature brand/product in YouTube video)

#### Food and Beverage Cue Display

Food and beverage cue display was coded in line with previous research (Scully et al., 2014; Holmberg et al., 2016) using the following categories; the context in which the food was presented, how the cue was described, how the influencer presented the cue, and the reason the cue was featured in the influencers video (see Table 1). A verbal reference was defined as a statement made in relation to a food or beverage product (e.g., “this is a good burger”) or brand name (e.g., “let’s go to McDonalds”), and did not include more general references made in relation to consumption (e.g., “I need to have lunch”).

### Statistical Analysis

To assess inter-rater coding reliability, a random 10% (*n* = 12 h) subset of videos were coded by an additional researcher and compared for consistency. Percentage agreement and a Cohen’s kappa analysis (chance agreement taken into account) were calculated. A percentage agreement of >80%, and a Cohen’s Kappa agreement of *k* > 0.60 were considered acceptable. For food cue type, percentage agreement was between 81.8 and 99.8%, Kappa agreement was between *k* = 0.80 and *k* = 0.85. For categories relating to food cue brand placement and presentation, all cues reached a percentage agreement between 80.2 and 99.7%, Kappa agreement between *k* = 0.61 and *k* = 0.80. Further analysis was performed using SPSS software (version 24 for Windows, SPSS Inc., Chicago, United States). Food cue categories were compared using Chi-squared tests and effect sizes calculated using Cramer’s *V*, with 0.04 indicating a small effect, 0.13 indicating a medium effect, and 0.22 indicating a large effect. Statistical significance was set at *p* < 0.05 and the exact values to *p* < 0.001 reported.

## Results

### Recording Statistics

In total 380 YouTube videos were analyzed, which equaled 119.5 h of content (female influencer = 47 h). Within the sample, there were 3571 food and beverage cues (*n* = 1092 female influencer), featured at an average rate of 29.9 cues per hour (female influencer *n* = 23.2, male influencer *n* = 34.2). A total of 27 videos (7.4%) did not feature any food or beverage cues.

### Nutrient Profiling

**FIGURE 1:**
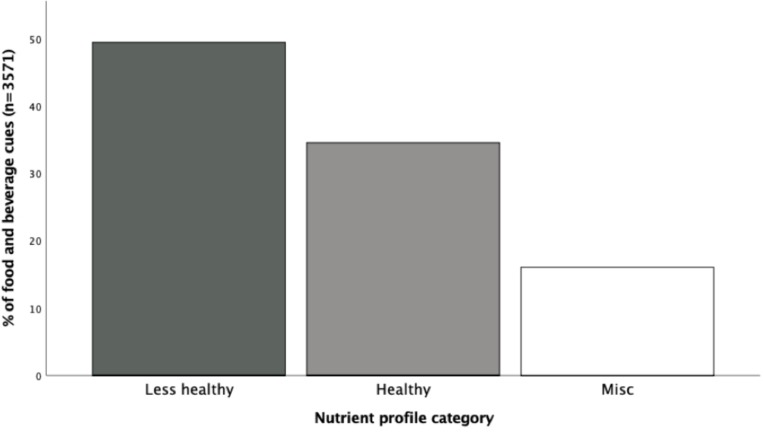
The percentage of food and beverage cues categorized by nutrient profile according to the UK Nutrient Profiling Model.

Food and beverage cues were categorized into three groups based on the UKNPM; healthy, less healthy, and miscellaneous (nutritional content not available, e.g., product not identifiable from the video). There was a significant difference between these categories (χ^2^(2) = 599.13, *p* < 0.001, *V* = 41). There was a greater prevalence and rate of less healthy cues (49.4%/14.8 per hour) than healthy (34.5%/10.3 per hour) or miscellaneous (16.1%/4.8 per hour) cues (see Figure 1).

### Food and Beverage Cue Type

Cues were categorized into 25 different product types (χ^2^(48) = 5088.75, *p* < 0.001, *V* = 0.84) (see Table 2). Cakes were the most frequently featured product (9.4%), followed by fast food (8.9%) and chocolate and confectionary (6.6%). Fruits (6.5%) and vegetables (5.8%) featured less frequently (see Table 3).

**TABLE 2:** Food and beverage types ordered by frequency of appearance in influencer YouTube videos.

**Food and beverage type**	**Frequency (*n* = 3571)**	**%**
Cakes	337	9.4
Fast food	319	8.9
Chocolate and confectionary	234	6.6
Fruit	233	6.5
High fatı/sugarı/salt spreads	219	6.1
Vegetables	208	5.8
Tea and coffee	202	5.7
Core foods combined	191	5.3
Water	170	4.8
Meat and meat alternatives	156	4.4
Alcohol	149	4.2
Full fat dairy	148	4.1
Breadı/riceı/potatoesı/noodles	145	4.1
Other	124	3.5
Unidentifiable food and drink	124	3.5
Ice creamsı/desserts	104	2.9
Sugar sweetened drinks	100	2.8
Snack foods	97	2.7
Fruit juice	90	2.5
Crumbedı/battered meat and meat alternatives	90	2.5
Low fat milkı/reduced fat milk and yogurt	42	1.2
Frozenı/fried potato products	39	1.1
Low sugarı/high fiber breakfast cereals	25	0.7
High sugarı/low fiber breakfast cereals	19	0.5
Artificially sweetened beverages	6	0.2

**TABLE 3:** Frequency of food and beverage cues in each presentation category, split by category according to the UK Nutrient Profiling Model (healthy, less healthy, or miscellaneous).

	**Overall (*n* = 3571)**	**Less healthy (*n* = 1765)**	**Healthy (*n* = 1233)**	**Misc (*n* = 573)**	**χ^2^**	**Cramer’s *V***
*Cue type (%)*					599.13^∗^	0.41
Food and beverages	3571(100)	1765(49.4)	1233(34.5)	573(16.1)		
*Brand*					206.06^∗^	0.17
Misc	455(12.7)	217(47.7)	155(34.1)	83(18.2)		
Branded	624(17.5)	413(66.2)	107(17.1)	104(16.7)		
Supermarket	156(4.4)	85(54.5)	63(40.4)	8(5.1)		
Unbranded	1914(53.6)	797(41.6)	822(42.9)	295(15.4)		
Food retail establishment	421(11.8)	253(60.1)	86(20.4)	82(19.5)		
*Cue context*					76.75^∗^	0.10
Supermarket	241(6.8)	123(51)	94(39)	24(10)		
Eating out	1437(40.3)	760(52.9)	398(27.7)	279(19.4)		
Home	1509(42.3)	696(46.1)	620(41.1)	193(12.8)		
Other	383(10.7)	186(48.6)	121(31.6)	76(19.8)		
*Cue description*					101.21^∗^	0.19
Positive	1728(48.4)	999(57.8)	505(29.2)	224(13)		
Negative	65(1.8)	30(46.2)	30(46.2)	5(7.7)		
Neutral	1778(49.8)	735(41.4)	697(39.3)	343(19.3)		
*Cue presentation*					112.82^∗^	0.17
Consumed and verbal reference	556(15.8)	333(58.8)	148(26.1)	85(15)		
Consumed, no verbal reference	220(6.2)	72(32.7)	79(35.9)	69(31.4)		
Not consumed and verbal reference	1662(46.5)	895(53.9)	550(33.1)	217(13.1)		
Not consumed, no verbal reference	1123(31.4)	465(41.1)	456(40.6)	202(18)		
*Reason cue was featured*					24.03^∗^	0.06
Not explicitly presented as part of a marketing campaign	3350(93.8)	1624(48.5)	1182(35.3)	544(16.2)		
Gifted by brand	200(5.6)	124(62)	47(23.5)	29(14.5)		
Paid by brand	21(0.6)	17(81)	4(19)	0(0)		

*Values given are frequencies. Percentages (%) in the overall column refer to the group% within each category. Percentages (%) in the less healthy, healthy, and miscellaneous columns refers to the% within the group. ^∗^*p* < 0.001.*

### Food and Beverage Cue Brand

There was a significant difference between food and beverage brand categories (χ^2^(8) = 206.06, *p* < 0.001, *V* = 0.17) (see Table 3). Overall, 53.6% of food and beverage cues were unbranded and 29.3% were branded (including food retail establishment brands). Healthy cues were slightly more likely to be unbranded than less healthy cues (42.9 vs. 41.6%), and less healthy cues were much more likely to be branded (66.2%) than healthy cues (17.1%).

### Food and Beverage Cue Display

There was a significant difference between the context in which food and beverages were displayed (χ^2^(6) = 76.75, *p* < 0.001, *V* = 0.10) (see Table 3). Overall, cues were marginally more frequently presented in the context of the home (42.3%), and least frequently in the context of “eating out” (40.3%). Less healthy cues were more frequently situated in the context of “eating out” (52.9%) compared with healthy cues (27.7%).

There was a significant difference between how food and beverage cues were described (χ^2^(4) = 101.21, *p* < 0.001, *V* = 0.19). Overall, 49.8% of cues were described neutrally, 48.4% positively (e.g., “it’s delicious”) and 1.8% negatively (e.g., “I don’t like it”). Less healthy cues were described more positively than healthy cues (57.8 vs. 29.2%) but there was no difference in how frequently they were described negatively (both 46.2%).

There was a significant difference between how food and beverages were presented in videos (χ^2^(6) = 112.82, *p* < 0.001, *V* = 0.17). Overall, it was more common for cues not to be consumed during the video, with (46.5%) or without (31.4%) a verbal reference (e.g., “let’s go to McDonalds”), than it was for cues to be consumed with (15.8%) or without (6.2%) a verbal reference. Less healthy cues were more frequently not consumed, with (53.9%) or without (41.1%) a verbal reference compared with healthy cues with (33.1%) or without a verbal reference (40.6%).

The reasons for why food and beverage cues were featured in the influencers’ videos significantly differed (χ^2^(4) = 24.03, *p* < 0.001, *V* = 0.06). Overall most cues were not explicitly presented as part of a marketing campaign (93.8%). A total of 5.6% of all cues were featured due to brands gifting or sending products to the influencer, and 0.6% were due to explicit paid marketing collaborations between brand and influencer. It was more common for less healthy cues to be featured as gifted (62%) or paid marketing (81%) compared with healthy cues (23.5 and 19%, respectively).

## Discussion

The current study analyzed YouTube video blogs of influencers popular with children to determine the extent and nature of food and beverage cues featured. The proportion of “healthy” and “less healthy” cues was determined using the UKNPM. Factors related to cue presentation were also explored. The results showed that almost all videos featured at least one food or beverage cue. “Less healthy” cues were more frequent than “healthy” cues. Overall, cues were mostly unbranded, presented in the context of the home, not consumed, and not explicitly presented as part of a marketing campaign. However, cues were presented in different contexts according to nutritional profile. Compared with healthy cues, less healthy cues were more often branded, presented in the context of eating out, described positively, and featured due to explicit marketing.

Food and beverage cues featured in a high proportion (92.6%) of the influencers’ videos, equivalent to 29.9 cues per hour. This is considerably higher than the rate previously found in studies of TV product placement (14.3 per hour) (Scully et al., 2014), TV advertisements (3.5 per hour) (Whalen et al., 2017) and advertisements (38%) featured in YouTube videos popular with children (Tan et al., 2018), but is consistent with the rate of Instagram posts which featured food and beverage cues shared by adolescents (85%) (Holmberg et al., 2016). The high prevalence of cues found in the current study, and Holmberg et al., is likely due to the nature of the content assessed. Food and beverages displayed in advertisements during TV programming, or in pop-up adverts in YouTube videos, are featured at specific intervals during the content being viewed. As a result, the rate of cues per hour is likely to be less compared with cues embedded in user-generated content, where exposure could potentially be continuous. In addition, user-generated content will feature various products that the user feels expresses themselves (Boyd and Ellison, 2007; Smith et al., 2012). For instance, YouTube video blogs can capture a user’s everyday life (Snelson, 2015; Lee and Watkins, 2016), and with food and beverages integrated into many social activities, it is highly likely that these cues will feature (Folkvord et al., 2019). If children feel as if they know an influencer on a personal level (Knoll and Matthes, 2017), and are trusting of their content (Ofcom, 2018), then high exposure to these cues could give children the impression that these items are important to an influencer which could impact their own relationship with food and beverages (Folkvord et al., 2019).

Of the UKNPM categories, “less healthy” food and beverages were featured most frequently (49.4%/14.8 cues per hour). This finding is consistent with the proportion of HFSS food and beverages found in TV advertising (Kelly et al., 2010, 2019; Boyland et al., 2011; Obesity Health Alliance, 2017; Whalen et al., 2017), product placement (Sutherland et al., 2010; Scully et al., 2014), websites popular with children (Kent and Pauzé, 2018) and social media (Holmberg et al., 2016; Tan et al., 2018; Folkvord et al., 2019; Potvin Kent et al., 2019). The prevalence of “less healthy” food and beverages in the current study may give children the impression that influencers regularly consume these items (Vassallo et al., 2018; Norwegian Consumer Council, 2019), and encourage similar behaviors (Martínez and Olsson, 2018). Indeed, previous research has showed that young people’s exposure to HFSS cues in social media, including via influencers content, increases attitudes toward, and immediate intake of, these products (Coary and Poor, 2016; Baldwin et al., 2018; Coates et al., 2019a; Folkvord et al., 2019). Although data on the amount of time that children in the United Kingdom spend watching YouTube video bloggers is not available, data from other countries enable an estimation of children’s potential exposure to “less healthy” food and beverage cues via this content. In the United Kingdom, each week 93% of 8–11 year old’s, and 99% of 12–15 year old’s, spend 13.5 and 20.5 h, respectively online, and roughly half of these children (44%/52%) report watching YouTube video bloggers (Ofcom, 2018; Revealing Reality, 2019). Self-report data from the Netherlands shows that on the days that children (10–13 years) view videos by their favorite YouTube video bloggers, 58% do so for less than 1 h per day, and 40% for more than 1 h per day (Folkvord et al., 2019). If those children in the United Kingdom, who watch YouTube video bloggers, spent an hour each day watching this content, it is estimated from the current study’s findings that they would be exposed to 104 “less healthy” food cues per week (14.8 cues per hour × 7), which equates to 5387 per year.

Healthier items accounted for just over a third (34.5%/4.8 cues per hour) of cues featured in influencer videos. This finding does not differ too substantially from that of a content analysis of images shared by adolescents on Instagram, where fruit and vegetables accounted for just over a fifth of all food and beverage cues shared (21.8%) (Holmberg et al., 2016). Notably, the proportion of healthy cues in user-generated content is higher than previously found in TV advertising, where studies have showed an almost complete lack of adverts for fruit or vegetables (Powell et al., 2013b; Whalen et al., 2017). The higher proportion of healthy items shared in user-generated content could be viewed positively from a health promotion perspective as this balance is more consistent with national dietary recommendations. With a few notable exceptions (including Harris et al., 2012), most previous studies that have explored the impact of digital marketing of healthy food and beverages on children’s immediate intake of these items found either no effect on intake, or a smaller effect than for less healthy items (Folkvord et al., 2013; Naderer et al., 2018; Coates et al., 2019a). It may be that children require more long-term exposure to these cues in order to see an effect on healthier food choices. Children report enjoying being part of a “follower” community on YouTube and view influencers as both role models and friends who provide support and advice (Ofcom, 2018). Therefore, children who are subscribed to influencers that regularly feature healthy food and beverages in their YouTube videos, and who have watched these videos for a long period of time, may well be affected by this content. Future research should seek to explore the impacts of this type of exposure on children’s attitudes toward healthy food and children’s health related behaviors over time. Given the variety of content now available to children through video sharing platforms, researchers may also wish to explore the impact on children of content promoting other health-related (e.g., physical activity) or pro-social (e.g., cooperation) behaviors.

Where food and beverages are purchased and consumed, and how this is communicated may play an important role in shaping the norms children develop around food (Best, 2014). Consumer lifestyle and contextual details are often communicated in HFSS marketing (Ginsberg, 2015; Vassallo et al., 2018). Details about *where* influencers eat as well as *what* they eat were regularly featured in the YouTube videos analyzed in the current study. Less healthy food and beverages were mainly consumed in the context of eating out (in coffee shops and fast food chains), whereas healthy food and beverages were consumed in the home, which is consistent with similar studies (Scully et al., 2014; Holmberg et al., 2016). The most frequently featured products, “cakes” (9.4%) and “fast food” (8.3%) were often purchased from these establishments, and many items in the “miscellaneous” category were beverages purchased in coffee shops. Although the contents of the beverage containers were not always apparent from influencer’s videos, the take-away lifestyle of the influencer was, which is a concern given that approximately 200 more calories are consumed per day by adults in the US (*n* = 12,528) when eating out of the home compared with in the home (Nguyen and Powell, 2014). In addition, data collected by the Food Standards Agency reveal that a significant proportion of the food United Kingdom adults consume is purchased from food retail establishments, and eating out is becoming more popular (Food Standards Agency, 2019). In 2010, 68% of adults reported that they had eaten out or bought a takeaway in the past week, compared with 75% in 2014 (Food Standards Agency, 2014). Therefore, it may be important to consider the impact of children’s exposure to an influencer’s take-away lifestyle on children’s food behavior norms.

With the rise of social media, details of users’ lives (including influencers and celebrities) are increasingly available, including information about the brands they consume (Click et al., 2013; Jin and Phua, 2014). In the current study, most of the featured food and beverage cues were unbranded, however, just under a third (29.3%, *n* = 1045) displayed a major brand name (including food retail establishment). In a similar content analysis, over half of the images shared by adolescents on Instagram featured food and beverage brands or logos (Holmberg et al., 2016). The frequency of brands displayed in both studies indicate that users may mimic brand focused techniques typically used in food and beverage marketing (Ginsberg, 2015; Vassallo et al., 2018). Brand-self connection refers to an individual’s identification with a brands characteristics, their own self-concept, and other brand users characteristics (Chaplin and Roedder John, 2005; Keller, 2009). Previous studies have found that children are more likely to prefer branded food items that they consider to be popular with others (Chaplin and Roedder John, 2005; Roper and La Niece, 2009), and that they appropriate brand meaning from brand endorsement by celebrities (Kelly et al., 2016; Escalas and Bettman, 2017) and influencers (Lee and Watkins, 2016; De Jans et al., 2018). Exposure to food and beverage brand marketing has been shown to have both brand specific (Borzekowski and Robinson, 2001; Robinson et al., 2007; Forman et al., 2009; Boyland et al., 2013) and category level effects on children’s food intake and preferences (Halford et al., 2008; Cairns et al., 2013). Therefore, it is likely that exposure to branded food and beverage cues via influencers’ YouTube videos has an impact on children’s food brand preferences as well as their short-term consumption.

Most of the food and beverage cues that featured in influencers’ videos were described positively, whereas just 1.8% of cues were described negatively. These findings are consistent with Holmberg et al. (2016) who found that 74.8% of user-generated Instagram images featuring food and beverages were captioned positively (e.g., smiley emojis), whereas just 1.3% were captioned negatively. Additionally, in the current study only 22% of food and beverages were actively consumed, again consistent with Holmberg et al. (2016) who found only 15.5% of images depicted partly consumed products. Social media users choose how they present themselves online (Blinka and Smahel, 2009), and so products and brands may be featured for self-representation rather than real-life consumption (Boyd and Ellison, 2007). For instance, a popular fast food meal may be featured in an influencers YouTube video for the purpose of creating content that is appealing to their audience, but in real life, the whole portion is not consumed. Media literacy is the ability to develop an informed critical understanding of the nature and impact of media content and is required in order to make judgements about the truthfulness of information (Mcginnis et al., 2006). Younger children’s (12 years and under) critical understanding of the commercial world is still evolving as part of their wider cognitive development (Story and French, 2004), and so they may be less likely than adults to apply critical thinking skills when online. In a previous study, young females (18–30 years) displayed the understanding that Instagram is rarely used to post negative reviews about products (Djafarova and Rushworth, 2017), and so are seemingly aware of the distinction between how social media users portray their lives online compared with reality. The distinction between how adults and children interpret food and beverage cues in social media influencer content would be worth exploring further.

A fundamental issue when regulating advertising on social media is whether or not the content is commercial in origin (Dunlop et al., 2016). In the current study, unless labeled with an advertising disclosure (e.g., #ad), influencers videos were assumed to not be financially driven by marketing budgets. The findings showed that food and beverage cues were more often not explicitly presented as part of a marketing campaign (93.8%), than brand payment (0.6%) or gifting (5.6%). Consistent with these results, celebrity-brand endorsements which appear as authentic and natural are increasingly common in social media (Marwick and Boyd, 2011) and have been shown to have a stronger persuasive effect than endorsements in advertising or product placement (Russell and Rasolofoarison, 2017). This is likely due to consumers brand evaluations being more positive when an endorser is perceived to be motivated by a products quality, rather than financial incentive (Bergkvist et al., 2016). The Reactivity to Embedded Food Cues in Advertising Model (REFCAM) states that the level of processing of a food cue influences the effect of exposure (Folkvord et al., 2016). In accordance with the REFCAM, cues that are featured due to natural influencer endorsements, compared with paid endorsements (e.g., #ad), are processed with less cognitive elaboration (Buijzen et al., 2010; Cauberghe and De Pelsmacker, 2010; Folkvord et al., 2016) meaning they are less likely recognized as advertising (Freeman et al., 2007; Rozendaal et al., 2011), and have a more persuasive effect (Folkvord et al., 2016). However, a previous study found that children (9–11 years) exposed to a YouTube video featuring influencer marketing of an HFSS snack (with and without an advertising disclosure) consumed more (kcals) of the marketed snack relative to an alternative snack (not featured in video), whereas children who viewed non-food marketing did not differ (Coates et al., 2019b). Thus, exposure to both natural and paid HFSS brand endorsements had an effect on children’s snack intake and brand preference. Future studies could explore qualitative differences in how children perceive food and beverage cues shared via user’s genuine recommendations compared with paid advertising, and if such perceptions influence health-related behavior.

Self-regulatory codes in the United Kingdom require that for influencer marketing where there is a financial relationship between the influencer and the brand, the content must be labeled with an advertising disclosure (Committee of Advertising Practice, 2017). Since completion of this study the rules were tightened, with items that are sent or “gifted” to influencers by brands now requiring a label (e.g., #gifted; Competition and Markets Authority, 2018). Additionally, caution should be taken in assuming that content does not feature marketing if it is not explicitly labeled as such (Potvin Kent et al., 2019). Over the past few years the ASA has warned 200–300 influencers about failure to comply with the rules (The Drum, 2019). The female influencer whose YouTube content was analyzed in the current study was one of sixteen high profile influencers who were investigated in 2019 by the Competition and Markets Authority for repeatedly breaking consumer law (BBC News, 2019). Therefore, it is likely that the current study substantially underestimates children’s exposure to items that are gifted to influencers, and if replicated the prevalence of explicit influencer marketing would increase.

Previous research in Norway showed approximately 20% of all influencer-brand marketing was for food and beverages (Norwegian Consumer Council, 2019). This marketing was considered to target younger viewers because of the popularity of the influencers with this demographic and the use of visual effects (fonts, emoji’s), humor, language and music being particularly appealing to a youth audience. At the time of writing, the influencers responsible for producing the content assessed in the current study collectively had just over 15 million YouTube subscribers (Social Blade, 2018). Using YouTube’s user demographics and the United Kingdom’s self-regulatory audience thresholds (ASA, 2017), approximately 3.75 million young people (13–17 years) could be exposed to HFSS product marketing by these particular influencers without any restrictions being applied. Also, this figure is likely to be a conservative estimate as YouTube’s demographics do not include children under the age of 13 who are known to be active on the platform (Ofcom, 2017). There are clear loopholes in the current regulation, and more effective rules that are appropriately monitored and enforced are required to enable children to participate in the digital world without their dietary health being adversely affected (WHO, 2016).

The current study has some limitations, but also many strengths. Firstly, influencers were selected based on country-wide popularity with children, not global popularity, which could limit the generalizability of findings. However, the borderless nature of the internet means that many children beyond the United Kingdom have access to these influencers’ YouTube videos. In addition, this study provides a methodological platform that could be replicated by other researchers to produce comparable multi-country data on this subject. Secondly, the video content of only two influencers was analyzed and the findings do not necessarily represent how food and beverage cues are featured by other influencers. However, it was paramount that we analyzed content that children are likely to be exposed to and which has been demonstrated to affect their eating behavior. It would be interesting for future research to analyze the video content of any child influencers popular with children, and to explore content on the YouTube Kids app to explore how food and beverages cues are featured there, and if there are any differences with the findings described here. Thirdly, cues that were deemed as not being prominent were not coded and so the findings may underrepresent the prevalence of food and beverages in this content. Finally, influencer content is only one of the many ways in which young people are exposed to food and beverage cues through YouTube, with adverts displayed before and during videos. However, analyzing the content of YouTube videos, as opposed to the advertising placed in and around them, overcomes issues of access to personalized content.

In conclusion, this study provides the first empirical assessment of the extent and nature of food and beverage cue presentation in YouTube video blogs by influencers popular with children. Foods and beverages were featured in almost all videos. “Less healthy” items (compared with “healthy”) were more frequently featured, likely to be branded, presented in the context of eating out, described positively, and featured as part of a marketing campaign. Given the emerging evidence of the effects of influencer marketing of food and beverages on children’s eating behavior, this study offers a useful methodological platform for digital food marketing assessment and delivers important contextual information about this evolving practice. Policymakers, social media platforms and influencers should consider how to implement real change in the food environment by limiting the widespread digital promotion of unhealthy food and beverages to young people.

## Data Availability Statement

The raw data supporting the conclusions of this manuscript will be made available by the authors, without undue reservation, to any qualified researcher.

## Author Contributions

AC and EB contributed to the conception and design of the study and wrote sections of the manuscript. AC organized the database, performed the statistical analysis, and wrote the first draft of the manuscript. AC, EB, CH, PC, and JH contributed to the manuscript revision, and read and approved the submitted version.

## Conflict of Interest

CH, PC, and JH report grants from American Beverage Association. CH and JH received personal fees from International Sweeteners Association, outside the submitted work. The remaining authors declare that the research was conducted in the absence of any commercial or financial relationships that could be construed as a potential conflict of interest.
